# The microvascular extracellular matrix in brains with Alzheimer’s disease neuropathologic change (ADNC) and cerebral amyloid angiopathy (CAA)

**DOI:** 10.1186/s12987-020-00219-y

**Published:** 2020-09-29

**Authors:** Mamatha Damodarasamy, Robert B. Vernon, Jasmine L. Pathan, C. Dirk Keene, Anthony J. Day, William A. Banks, May J. Reed

**Affiliations:** 1 Division of Gerontology and Geriatric Medicine, Department of Medicine, University of Washington, Seattle, WA USA; 2 Geriatric Research Education and Clinical Center, VA Puget Sound Health Care System, Seattle, WA USA; 3 Matrix Biology Program, Benaroya Research Institute at Virginia Mason, Seattle, WA USA; 4 Division of Neuropathology, Department of Pathology, University of Washington, Seattle, WA USA; 5 Wellcome Trust Centre for Cell-Matrix Research and Lydia Becker Institute of Immunology and Inflammation, Division of Cell-Matrix Biology & Regenerative Medicine, School of Biological Sciences, Faculty of Biology, Medicine and Health, University of Manchester, Manchester Academic Health Sciences Centre, Manchester, UK; 6 Division of Gerontology and Geriatric Medicine, Department of Medicine, University of Washington Harborview Medical Center, Seattle, WA 98104 USA

**Keywords:** Human neuropathology, Alzheimer’s disease, Cerebral amyloid angiopathy, Vascular density, Vascular diameter, Laminin, Collagen IV, Fibronectin, Perlecan, TSG-6, Hyaluronan

## Abstract

**Background:**

The microvasculature (MV) of brains with Alzheimer’s disease neuropathologic change (ADNC) and cerebral amyloid angiopathy (CAA), in the absence of concurrent pathologies (e.g., infarctions, Lewy bodies), is incompletely understood.

**Objective:**

To analyze microvascular density, diameter and extracellular matrix (ECM) content in association with ADNC and CAA.

**Methods:**

We examined samples of cerebral cortex and isolated brain microvasculature (MV) from subjects with the National Institute on Aging-Alzheimer's Association (NIA-AA) designations of not-, intermediate-, or high ADNC and from subjects with no CAA and moderate-severe CAA. Cases for all groups were selected with no major (territorial) strokes, ≤ 1 microinfarct in screening sections, and no Lewy body pathology. MV density and diameter were measured from cortical brain sections. Levels of basement membrane (BM) ECM components, the protein product of TNF-stimulated gene-6 (TSG-6), and the ubiquitous glycosaminoglycan hyaluronan (HA) were assayed by western blots or HA ELISA of MV lysates.

**Results:**

We found no significant changes in MV density or diameter among any of the groups. Levels of BM laminin and collagen IV (col IV) were lower in MV isolated from the high ADNC vs. not-ADNC groups. In contrast, BM laminin was significantly higher in MV from the moderate-severe CAA vs. the no CAA groups. TSG-6 and HA content were higher in the presence of both high ADNC and CAA, whereas levels of BM fibronectin and perlecan were similar among all groups.

**Conclusions:**

Cortical MV density and diameter are not appreciably altered by ADNC or CAA. TSG-6 and HA are increased in both ADNC and CAA, with laminin and col IV decreased in the BM of high ADNC, but laminin increased in moderate-severe CAA. These results show that changes in the ECM occur in AD and CAA, but independently of one another, and likely reflect on the regional functioning of the brain microvasculature.

## Background

Alzheimer’s disease (AD) neuropathologic change (ADNC) is defined by histologic criteria of amyloid-β (Aβ) and tau deposition throughout brain regions, which are validated and subject to periodic re-evaluation [1, 2]. Cerebral amyloid angiopathy (CAA), a related disease that is also characterized by established histologic criteria, refers specifically to the deposition of Aβ in the media and adventitia of small and mid-sized arteries (and, much less frequently, veins) of the cerebral cortex and the leptomeninges. In most cases of CAA there is some level of ADNC, but the converse is not true, since many brains with ADNC do not have significant CAA. This distinction has clinical significance in that CAA is an independent risk factor for microaneuryms and intraparenchymal hemorrhages [3, 4].

Study of the microvasculature in aging is complicated by reductions in vessel density (thought due to rarefaction), a thickening of the vascular wall that reflects changes in basement membrane (BM) structure, and poorly characterized alterations in the glycocalyx [5–8]. Consequently, attribution of changes in brain microvasculature (MV) specifically to ADNC or CAA is confounded by the associated factors of advanced age, and other neuropathology that increases with aging such as Lewy bodies, strokes, and microinfarcts [9, 10]. As a result, studies focusing specifically on the vascular changes in AD and CAA have generated conflicting results with respect to blood vessel density, morphology, and associated extracellular matrix (ECM) [11, 12].

The BM of brain MV is an ECM of unique composition at the abluminal surface of vascular endothelial cells, which provides a dynamic interface for cell-ECM communication. The thickness of the normal BM is approximately 40–100 nm and consists of at least four major ECM proteins: laminin, collagen IV (col IV), fibronectin, and nidogen [13]. Also present in the BM are heparan sulfate proteoglycans (HSPGs), such as perlecan and agrin. Laminin and col IV are the dominant components in terms of content and provision of structural support. BM organization begins with deposition of laminin, which interacts with cells (primarily via integrins) to polymerize and bind to less highly expressed ECM components, such as perlecan and fibronectin. The final steps in BM assembly are mediated primarily by col IV, a highly expressed collagen that becomes organized into a 3-dimensional network that stabilizes the structure of the BM [14]. Previous studies of changes in BM ECM in AD and CAA neuropathology have yielded variable results in human and animal models [15–18].

Hyaluronan (HA) is a ubiquitous non-sulfated glycosaminoglycan (GAG) that is constitutively secreted as a large linear polymer throughout the vascular glycocalyx and the gray and white matter of the brain parenchyma [13]. HA synthesis and turnover is accelerated during injury, resulting in greater amounts of lower MW HA [19]. Whereas high molecular weight HA binds to the highly expressed CD44 receptor, smaller forms of HA are effective competitors of these interactions [20, 21]. It has also been reported that low molecular weight HA interacts directly with CD44 or toll-like receptors to promote inflammation [22–25]; however, at least some of these effects have been attributed to lipopolysaccharide (LPS) contamination in the HA preparations used [26]. HA is usually present in complex with HA-binding proteins (hyaladherins) that together play a critical role in tissue organization and structure; e.g. such as the specialized glycocalyx of perineuronal nets [21]. One such hyaladherin, TSG-6, while only expressed in response to inflammation in most tissues, is constitutively present in the brain ECM [27, 28]. In this context, TSG-6 likely organizes HA molecules into a crosslinked network and plays a protective role in response to brain injury/inflammation [27, 29, 30]. Our previous studies have found greater amounts of HA and TSG-6 in brains with high ADNC relative to those with no ADNC [31].

We wished to determine whether the density, morphology, and ECM content of MV differ in cortical brain tissues with advanced ADNC or CAA. In the present study, we compared age-matched human brain samples, lacking or with varying degrees of either ADNC and CAA, with respect to MV density, MV diameter, and MV ECM composition. AD brain samples were obtained from the parietal cortex of human subjects with not-, intermediate-, or high ADNC. Brain samples with CAA were obtained from the occipital cortex of human subjects divided into two groups: no CAA/not AD and moderate-severe CAA/intermediate-high AD. None of the specimens had significant concurrent vascular or Lewy body neuropathology.

## Methods

### Brain tissue samples

Samples of human brain tissue were obtained from the tissue repository of the University of Washington Neuropathology (UW NP) Core, where fresh samples are collected within 8 h of death (post-mortem interval = PMI) and rapidly preserved by flash freezing in liquid N_2_. Non-frozen samples from short PMI subjects, and all tissues from subjects with > 8 h PMI, were fixed in 10% neutral-buffered formalin, sampled, processed, and then embedded in paraffin (formalin-fixed, paraffin-embedded, FFPE) according to routine protocols (Table 1).

**Table 1:** Samples for IHC {paraffin embedded} and biochemistry [frozen]

	AD brain samples		
	Not AD	Int AD	High AD
Female samples	{8} [4]	{7} [5]	{23} [19]
Mean age (range)	81 (71–88)	86 (72–92)	88 (70–99)
Male samples	{13} [8]	{11} [8]	{17} [13]
Mean age (range)	85 (73–95)	89 (77–100)	83 (67–96)

	CAA brain samples		
	No CAA	Mod-Severe CAA	
Female samples	{7} [7]	{14} [14]	
Mean age (range)	86 (77–98)	81 (59–98)	
Male samples	{7} [7]	{12} [12]	
Mean age (range)	85 (64–101)	82 (43–96)	

Table shows sex and age distribution for samples available for histology {paraffin embedded} and biochemistry [frozen] at the time of death

*Int AD*= Intermediate AD, *Mod-Severe CAA*= moderate-severe CAA

Two groups were studied: one set was comprised of parietal lobe samples that focused on AD pathology (“AD samples”), whereas the second set consisted of occipital lobe samples focused on CAA pathology (“CAA samples”). For the AD samples, we chose to examine parietal cortex (inferior parietal lobule) as a region exemplifying AD pathologic progression by Aβ plaques and neurofibrillary tangles. Cases were selected based on the level of ADNC for neuritic plaque density (CERAD score) and neurofibrillary tangle distribution (Braak stage) in subjects with no Lewy body pathology and no more than 1 microinfarct in standard screening sections [2]. Graded levels of Aβ and tau pathology in parietal cortex samples were confirmed by a board-certified neuropathologist at the original autopsy, using an adaptation of NIA-AA guidelines for CERAD neuritic plaque density and Braak stage for neurofibrillary tangle distribution as assessed by Bielschowsky silver stain and immunostains for hyperphosphorylated tau (pTau), respectively [32]. Since Thal phase was not available for all research participants, levels of ADNC in the samples were classified as “not ADNC” (CERAD = 0, Braak = 0–II, *n* = 21 FFPE/12 frozen); “intermediate ADNC” (CERAD = 2, Braak = III–IV, *n* = 18 FFPE/13 frozen); and “high ADNC” (CERAD = 3, Braak = V–VI, *n* = 40 FFPE/32 frozen). Female/male representation was 38%/62% (not ADNC), 39%/61% (intermediate ADNC) and 57%/43% (high ADNC), with an age range of 71–95 years (not ADNC), 72–100 years (intermediate ADNC) and 67–99 years (high ADNC). Patients in the high ADNC group did not differ significantly from those in the not ADNC group with respect to age, but there were more females than males in the high ADNC group and more males than females in the not ADNC group (Table 1).

CAA pathology is common in the occipital lobe [3] and historically is graded in this region in the UW NP Core. Specifically, using histochemical and immunohistochemical assessments, CAA is judged as absent with no labeling, mild if the CAA is limited to leptomeningeal vessels, moderate if a minority of penetrating cortical arterioles are positive, and severe if a majority of penetrating arterioles are involved. Accordingly, for the CAA-focused portion of the study we chose to examine occipital cortex as neuropathologic assessments for CAA were performed in these tissue samples, and this is a region well-suited to capturing the neuropathologic changes of CAA [9]. Cases were selected based on the level of CAA in subjects with no Lewy body disease and no more than 1 microinfarct. Since CAA neuropathology is usually accompanied by ADNC, levels of CAA and ADNC in the CAA samples were classified (as follows): “no CAA/not ADNC” (*n* = 14 FFPE/14 frozen); “moderate-severe CAA/intermediate-high ADNC” (*n* = 26 FFPE/26 frozen) using an adaptation of, and in alignment with, NIA-AA guidelines [32]. Female/male representation was 50% in the CAA groups with an age range of 64–101 years (no CAA group) and 59–98 (moderate-severe CAA group). Patients in the moderate-severe CAA group did not differ significantly from those in the no CAA group with respect to sex or age (Table 1).

### Immunohistochemistry (IHC)

The FFPE brain tissue samples described above were sectioned at 5 µm, mounted on slides, deparaffinized, the antigens unmasked by heating to 100 °C in pH 6.0 citrate buffer (Item 3300, Vector Laboratories, Burlingame, CA), and endogenous peroxidases inactivated with 3% (v/v) H_2_O_2_ in Tris-buffered saline. The sections were then blocked in 2% (v/v) normal goat serum. Subsequently, the sections were exposed to 1–5 µg/mL of primary rabbit polyclonal antibodies (pAbs—all from Abcam, Cambridge, UK) that recognized human alpha-smooth muscle actin (Item ab5694), PECAM/CD31 (Item ab28364), or von Willebrand factor (vWF) (Item ab6994). The latter two are specific markers for endothelial cells [33, 34]. Bound anti-alpha-smooth muscle actin pAb was visualized by immunofluorescence (IF), using Alexa-Fluor 594-goat anti-rabbit IgG (Item A11012, Thermo Fisher Scientific, Waltham, MA) in conjunction with 4′,6-diamidino-2-phenylindole (DAPI) nuclear stain. Bound pAbs to vWF and PECAM were visualized chromogenically using biotinylated secondary antibodies in conjunction with Vectastain^®^ Avidin–Biotin Complex (ABC) (Item PK-6105, Vector) and 3,3′-diaminobenzidine (DAB) (Item SK-4105, Vector). Cell nuclei were counterstained with hematoxylin. Labeled sections of the gray matter of the cortex were randomly selected, with no overlap, and imaged by a blinded observer with a Leica DMR epifluorescence microscope (Leica Microsystems, Wetzlar, Germany) equipped with a SPOT™ 1.4 megapixel CCD camera (Diagnostic Instruments, Sterling Heights, MI) (for IF) and a Leica DM2500 microscope equipped with a SPOT Insight 4 megapixel color CCD camera (for brightfield).

### Quantification of labeled brain sections

Paraffin sections representing each of the groups described in “Brain tissue samples” above were stained at the same time and under the same conditions using identical exposure parameters. MV density from digital photomicrographs (4 images per section) was quantified using ImageJ (National Institutes of Health image analysis freeware, https://imagej.nih.gov/ij/). Quantification was expressed as an “area fraction”; i.e., the area in µm^2^ stained with MV markers (alpha-smooth muscle actin, PECAM, or vWF) divided by the area of the “standard” photomicrographic field (4 mm^2^) × 100% [35]. Measurement of MV luminal diameter (in µm) was performed by collecting digital images at 10× magnification and measuring 4 vessels within each image (3 images per section).

### Isolation of brain MV

Frozen samples of parietal cortex or occipital cortex (without meninges) were thawed and the grey matter dissected away from white matter. For each sample, the isolated grey matter was triturated in serum-free Dulbecco’s Modified Eagle Medium (DMEM) with 4–5 strokes of a Dounce-type tissue grinder with a Teflon plunger. The triturated samples were centrifuged (2000×*g*) at room temperature for 15 s, the supernatants discarded, and the vascular pellet was resuspended in DMEM. When necessary to remove large fragments and single cells, the suspension was filtered through 300 µm and 30 µm meshes, respectively. To eliminate debris: Dextran gradient isolation was done using 40% (w/v) dextran in isolation buffer (DMEM low glucose with 1% Dextran) followed by centrifugation at 3000×*g* for 15 min at 4 °C. Retentates were evaluated by phase contrast microscopy to confirm the presence of MV. The samples were then washed 3 times by centrifugation through cold PBS and either imaged by IF or lysed for polyacrylamide gel electrophoresis (PAGE)/western blot analysis (described below).

### MV imaging by IF

A subset of isolated MV was fixed in neutral-buffered formalin, blocked in 2% goat serum, and exposed to rabbit pAbs recognizing human PECAM (Item ab28364, Abcam), vWF (Item ab6994, Abcam), PDGFRbeta (Item 3169S, Cell Signaling Technology), GFAP (Item ab7260, ABCAM), laminin (Item L9393, Millipore-Sigma, Burlington, MA), or col IV (Item ab6586, Abcam). Bound pAbs were visualized using Alexa-Fluor 594-goat anti-rabbit IgG. Cell nuclei were counterstained with DAPI. Labeled sections were imaged with the Leica DMR microscope/SPOT camera system described previously.

### PAGE/western blot immunoassays

Isolated MV were lysed in M-PER™ Mammalian Protein Extraction Reagent (Item78505, ThermoFisher) with protease inhibitors (Item78439, ThermoFisher). Total protein content of the lysates was measured by bicinchonic acid assay. A portion (20 µg total protein) of each sample was reduced using NuPAGE™ Sample Reducing Agent (ThermoFisher), resolved by PAGE, transferred to nitrocellulose, and probed with 2–5 µg/mL of primary antibody as previously described [36]. Antibodies used were rabbit pAbs recognizing laminin (Item L9393, Millipore-Sigma), col IV (Item ab6586, Abcam), fibronectin (Item AB2033, Millipore-Sigma), or TSG-6 (RAH-1; [37]). A rat monoclonal antibody recognizing human HSPG2 (perlecan) (Item ab2501, Abcam) was also used. Primary antibodies bound to the blots were visualized with the appropriate horseradish peroxidase‐conjugated secondary antibodies (Jackson Immuno Research, West Grove, PA) (1 µg/mL), visualized by enhanced chemiluminescence (GE Healthcare Life Sciences, Pittsburgh, PA), scanned, and quantified using ImageJ as previously described [36].

### HA ELISA

MV isolates were washed once with PBS and homogenized in PBS with a tissue homogenizer. An equal volume of Cell Lysis Buffer 2 (Item 895347, R&D Systems/Bio-Techne) was added and tissues were lysed. Debris was then removed by centrifugation. An aliquot of the lysate was removed and assayed to determine HA levels using a quantitative sandwich enzyme immunoassay technique with a Hyaluronan Quantikine ELISA kit (R&D Systems) per the manufacturer’s instructions. HA content in the samples was normalized to the total protein content of each sample.

### Statistical analysis

Initial comparisons for significant differences among not, intermediate and high ADNC for MV density were made by ANOVA. Significant differences between not versus high ADNC and no CAA versus moderate-severe CAA for MV density, basement membrane components, TSG-6, and HA content were then determined using a paired Student’s t-test with unequal variance. Statistical significance was defined as p < 0.05.

## Results

The focus of this study was to characterize changes in MV density, MV diameter, and levels of key ECM components in brain samples exhibiting differing levels of ADNC or CAA neuropathology. Samples from the parietal lobe were selected to assess ADNC, as described in “Methods”. Samples from the occipital lobe were selected to assess CAA, as described in “Methods”. Grading was confirmed by a board-certified neuropathologist at the original autopsy, using appropriate criteria.

**Fig. 1:**
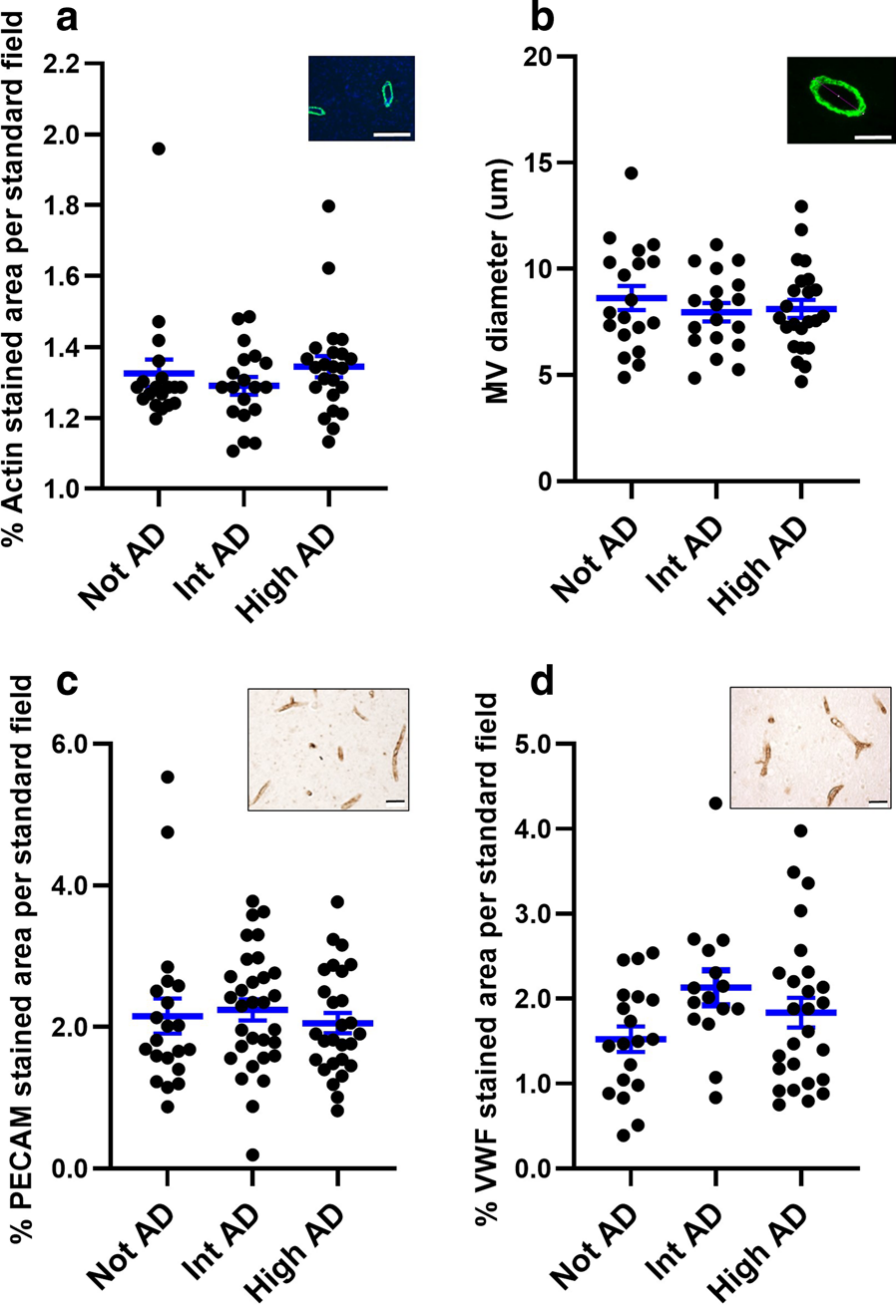
Density and diameter of MV are not significantly different in samples of parietal cortex from not, intermediate, and high ADNC subjects. Density (**a**) and diameter (**b**) of MV (identified by IF for alpha-smooth muscle actin as shown in insets of representative images in both **a** and **b**) in sections of parietal cortex in not ADNC, intermediate (Int) ADNC, and high ADNC. **c** MV density assays similar to those in **a**, but identifying MV by chromogenic IHC for endothelial cell PECAM (**c**, scatter plot with mean and SEM; inset, representative image) and vWF (**d**, scatter plot with mean and SEM; inset, representative image). These assays show no significant differences in MV density and diameter among the three groups. Inset scale bars are 20 µm (**a**, **c**,** d**) and 10 µm (**b**). Sample sizes (*n*) are indicated in “Results”

The cortical thickness of the specimens was measured to confirm that there were no significant differences in cortical atrophy among the samples obtained from each of the groups (data not shown). Initial experiments examined the organization of MV in sections from the AD brain samples (not ADNC, intermediate ADNC, or high ADNC groups). Analysis of MV by IHC was limited to areas at least 100 µm from an amyloid plaque. Alpha-smooth muscle actin staining of microvasculature showed that there were no significant differences in MV density (Fig. 1a) or diameter (Fig. 1b) among the three groups (*n* = 19 not ADNC, *n* = 15 intermediate ADNC, and *n* = 23 high ADNC). IHC for endothelial cell PECAM (Fig. 1c) and vWF (Fig. 1d) to mark MV also revealed no differences in MV density among the groups (for PECAM staining: *n* = 19 not ADNC, *n* = 15 intermediate ADNC, *n* = 39 high ADNC; for vWF staining: *n* = 10 not ADNC, *n* = 9 intermediate ADNC, *n* = 17 high ADNC). In parallel experiments, we found no significant differences in MV density in frontal lobe cortex samples from not-, intermediate-, and high ADNC groups, as assayed by IHC for alpha-smooth muscle actin (*n* = 5 for each group) and PECAM (*n* = 10 for each group) (data not shown).

**Fig. 2:**
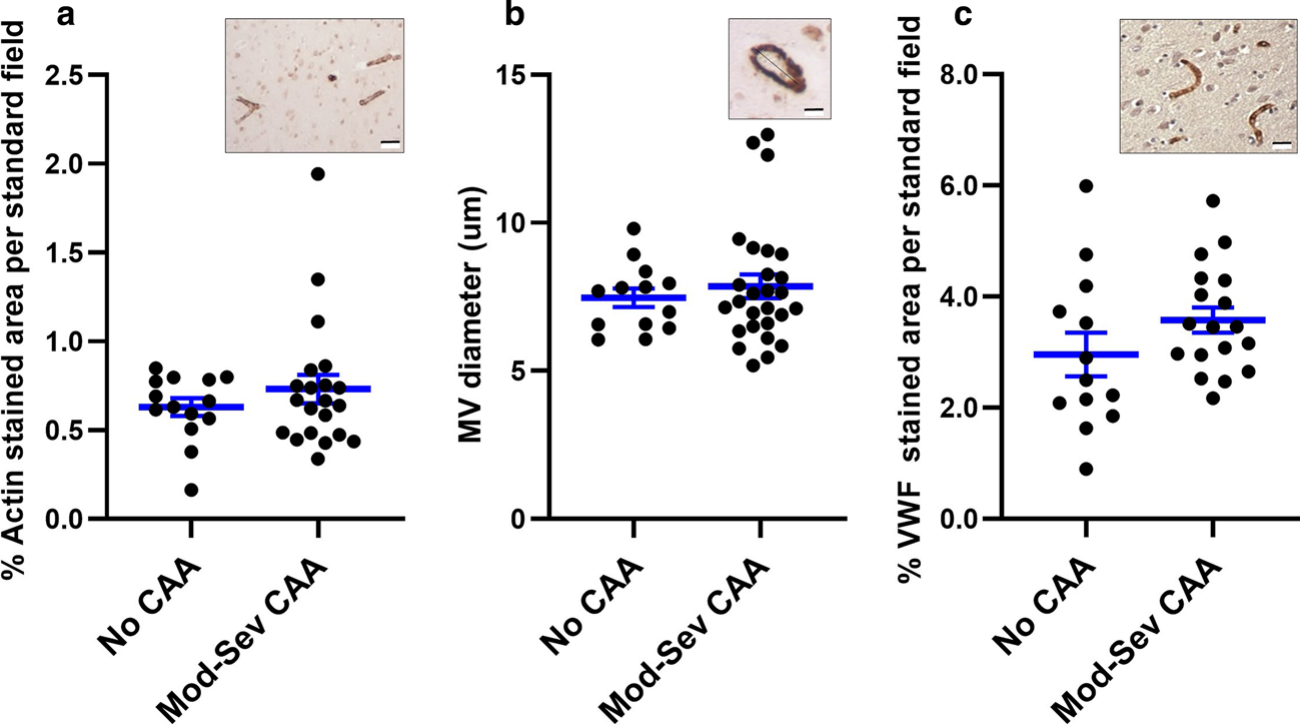
Density and diameter of MV are not significantly different in samples of occipital cortex with no and moderate-severe CAA pathology. Shown are scatter plots with mean and SEM. Density (**a**, **c**) and diameter (**b**) of MV were measured on samples labeled by IHC for alpha-smooth muscle actin (**a**, **b** with insets of representative images) and vWF (**c** with inset of representative image). These assays show no significant differences in MV density and diameter among the three groups. Inset scale bars are 20 µm (**a**, **c**) and 5 µm (**b**). Sample sizes (*n*) are indicated in “Results”

Similar assays were performed on sections from the no CAA and moderate-severe CAA groups. Alpha-smooth muscle actin staining showed that there were no significant differences in MV density (Fig. 2a) or diameter (Fig. 2b) between the groups (*n* = 13 no CAA *, n* = 22 moderate-severe CAA). IHC for VWF (Fig. 2c) to mark MV also revealed no differences in MV density among the groups (*n* = 13 no CAA and *n* = 22 moderate-severe CAA).

**Fig. 3:**
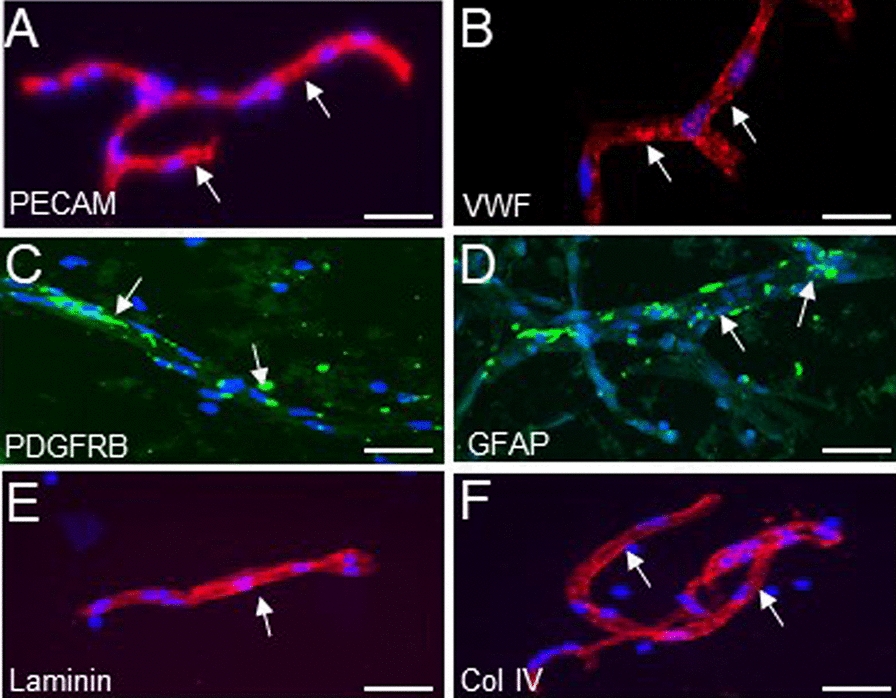
IF labeling (red or green stain) of isolated brain MV for markers of endothelial cells, pericytes, astrocytes, and components of BM ECM. MV were isolated from their respective regions of the cerebral cortex. Representative images show MV segments with endothelial cells positive for PECAM (**a**), vWF (**b**), PDGFRB (**c**), GFAP (**d**). MV segments also retain the BM components laminin (**e**) and col IV (**f**). Cell nuclei are labeled with DAPI (blue). Scale bars in **a**–**f** are 50 µm

In subsequent experiments, we assessed the relative expression of major BM components of MV isolated from frozen AD or CAA samples (each frozen sample had a corresponding FFPE sample that had been assessed by IHC). The isolated MV were confirmed to be vasculature by morphology and by IF assay for expression of PECAM (Fig. 3a) and VWF (Fig. 3b). The MV contained components of pericytes (Fig. 3c) and astrocytes (Fig. 3d) as well as the BM, as shown by IF for laminin (Fig. 3e) and col IV (Fig. 3f).

**Fig. 4:**
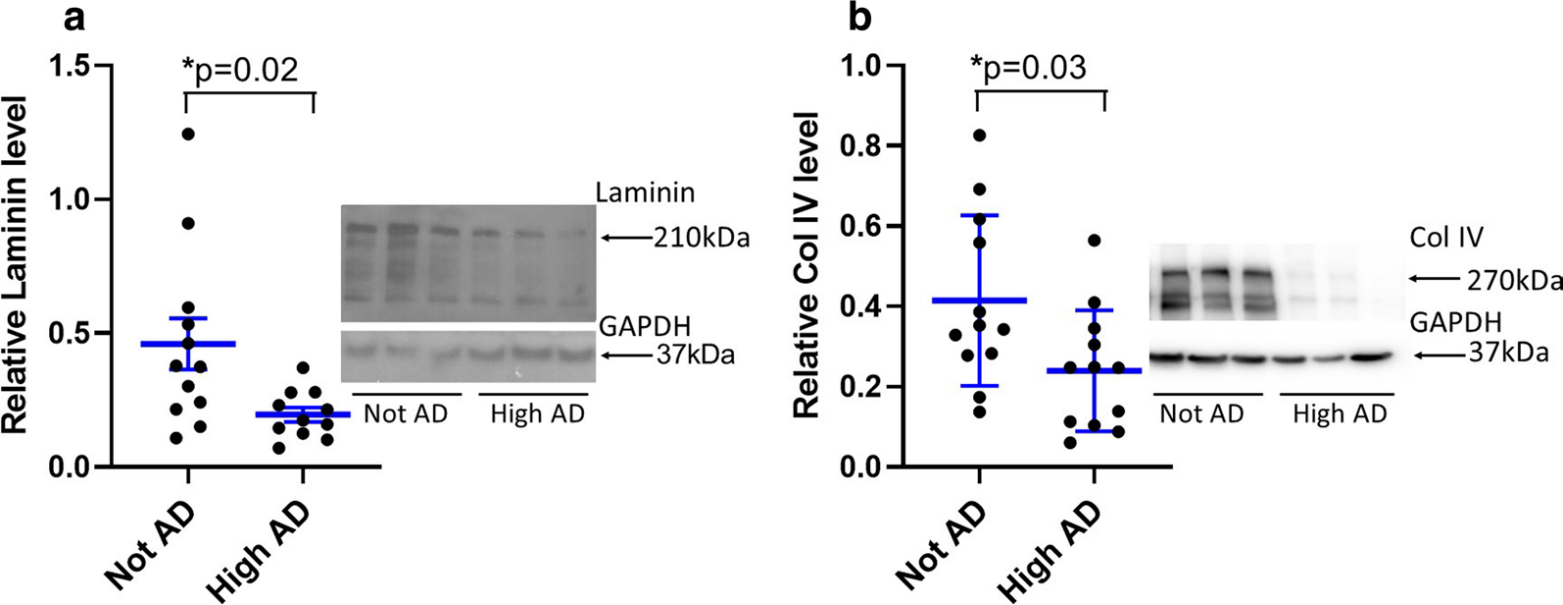
Differential content of laminin and col IV of MV isolated from not ADNC vs. high ADNC tissues. Relative levels of laminin and col IV to GAPDH were measured by western immunoblot assay of lysates of MV isolated from not ADNC and high ADNC groups. There were significant decreases in laminin (**a**, inset of representative western blot) and col IV (**b**, inset of representative western blot) in the high ADNC group vs. not ADNC group. Shown are scatter plots with mean and SEM, p values are as shown. Sample sizes (*n*) are indicated in “Results”

The isolated MV from the AD and CAA brain samples were then subjected to PAGE/western blot immunoassay and HA ELISA to assess the levels of key ECM components. With respect to the AD samples (obtained from the parietal lobe), the ECM proteins laminin (Fig. 4a) and col IV (Fig. 4b) were significantly lower in MV from high ADNC brains relative to not ADNC brains (*n* = 12 per group, laminin *p* = 0.02 and col IV *p* = 0.03). In contrast, the expression of fibronectin and perlecan were not significantly different between the high ADNC and not ADNC groups (data not shown). We have previously shown that high ADNC samples have significantly higher TSG-6 and HA content relative to not ADNC samples [31].

**Fig. 5:**
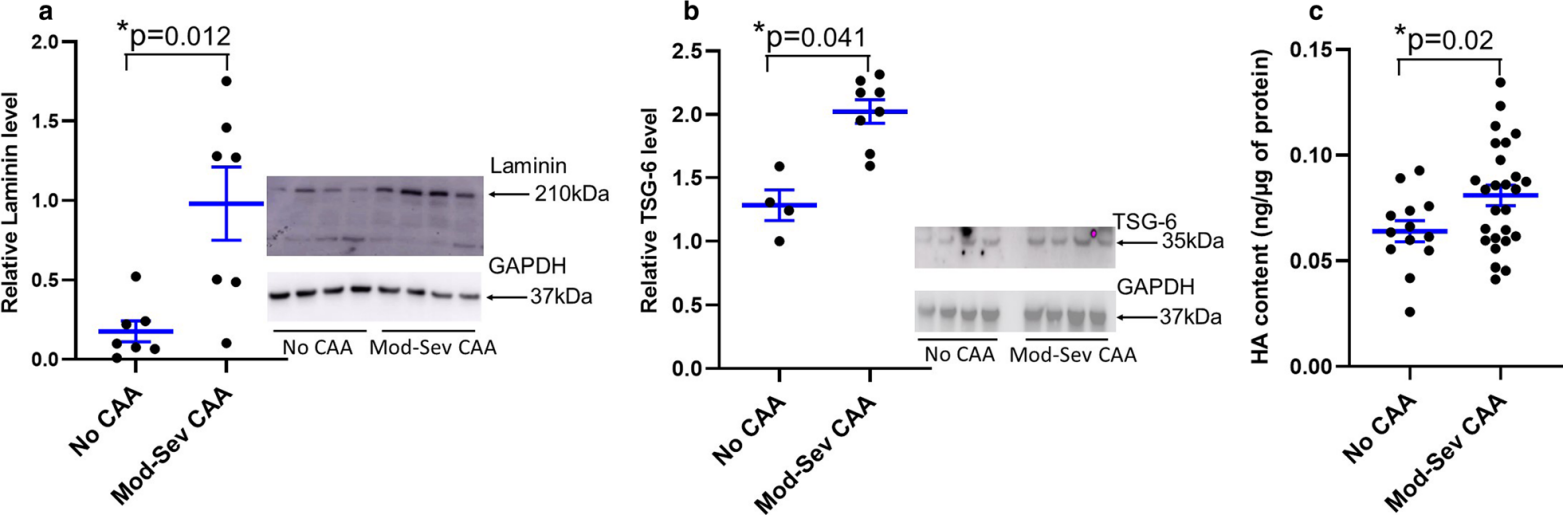
Differential content of laminin, TSG-6, and HA in MV isolated from no CAA and moderate to severe CAA brains. Western immunoblot assays showed that expression of laminin (**a**, inset of representative western blot) was significantly lower in lysates of MV isolated from no CAA brains vs. moderate-severe (Mod-Sev) CAA brains). Analyses of TSG-6 (**b**, inset of representative western blot) and HA (ELISA) content showed significant increases with moderate to severe CAA. Shown are scatter plots with mean and SEM, p values are as shown. Sample sizes (*n*) are indicated in “Results”

In studies of the CAA samples (no CAA, *n* = 7; moderate-severe CAA, *n* = 7), expression of laminin (Fig. 5a) was significantly higher in MV from moderate-severe CAA brains vs. the no CAA brains (*p* = 0.012)—a trend that was opposite to that seen in the ADNC samples, where laminin expression decreased coincidently with ADNC (Fig. 4a). The decrease in expression of laminin was supported by studies with a specific antibody against alpha 5 laminin (Invitrogen MA5-24649, data not shown). With the number of samples available, we were not able to detect differences in col IV, fibronectin, or perlecan (data not shown). However, TSG-6 (Fig. 5b) and HA (Fig. 5c) levels were both significantly higher in mod-severe CAA brains (*n* = 8 for western blot and *n* = 26 for HA ELISA) vs no CAA brains (*n* = 4 for western blot and *n* = 14 for HA ELISA), which was similar to what we have previously found in high ADNC vs not ADNC [31].

## Discussion

The microvasculature of the brain is a dynamic network that regulates blood flow to meet the high metabolic needs of neurons. Prior studies measuring AD-associated changes in MV density or morphology reported increased MV density in AD brains relative to age-matched control brains [11, 38, 39], but other studies have reported a lower MV density in AD brains compared to control brains [40]. Studies of changes in MV in CAA brains are not as plentiful as corresponding studies of AD, and the CAA studies also report conflicting data [3, 16]. Analysis of the status of MV in CAA is further complicated by the fact that CAA pathology varies by brain region in a manner that does not always correlate with the pathologic changes of AD. In contrast to previous studies, our data indicate that in the absence of chronic vascular brain injury and Lewy body pathology, there are no significant differences in MV density or diameter in parietal or frontal cortex tissues with high ADNC relative to intermediate AD and not ADNC brain samples. Similarly, MV density and diameter do not differ in occipital cortex of brains with moderate-severe CAA vs. no CAA. Our results reflect analyses of three different markers of MV. The conflicting data reported by prior studies could reflect, in part, the impact of cortical atrophy, which can be seen in association with a variety of pathologies including AD and vascular brain injury. Loss of brain volume due to atrophy, which is difficult to measure in isolated portions of brain samples, could mask a decrease in MV density. Additional variability in measures of MV density could arise in areas of inflammation, such as near amyloid plaques. Both of these variables were accounted for in our analyses.

The BM of the MV contributes to the integrity of the blood–brain barrier (BBB) [13]. The BBB (comprised of brain endothelial cells tightly associated with pericytes, astrocyte end feet, the glycocalyx, and the BM) restricts the passage of intravascular contents into the brain parenchyma [14, 41–43]. The BM is sometimes described as having endothelial and parenchymal layers, which are constituted from sequential deposition of structural and regulatory ECM [13, 44]. The ECM responds to forces from both the luminal and abluminal (parenchymal) side via integrins and other ECM-binding cell surface receptors. As a consequence, it is generally accepted that BM thickening, largely due to changes in laminin and col IV, occurs in the microvasculature throughout the body in response to chronic stresses, such as shear forces, high pressures, and aging [7, 16, 45, 46]. Accordingly, it is reasonable to predict that the thickness and ECM composition of the BM would change in brain MV in the presence of high ADNC or severe CAA neuropathology.

In the present study, we found that laminin was significantly lower in lysates of MV isolated from high ADNC vs. not ADNC brains. Our data are based on what is generally considered a pan laminin antibody [47, 48], but few prior studies have specifically examined laminin in the BBB with neurodegeneration. Early work reported an increase in punctate deposits of α1 and γ1 laminin chains in plaques of Aβ and in astrocytes of human brain parenchyma [49]. In IHC studies of Braak stage III–VI AD patients relative to age-matched controls, laminin exhibited patterns indicating BM fragmentation in AD and co-localized with Aβ in senile plaques, which could reflect a net decrease in intact laminin with AD [50]. We also found that col IV was lower in MV lysates from high ADNC vs. not ADNC tissues. Other studies of changes in col IV with AD have shown variable results. Early studies reported a 55% increase in col IV content in the cerebral MV of AD patients compared to controls [51]. Additional investigations of the brains of AD patients have reported a thickening of the BM together with an increase in collagen content [52–55]. Moreover, a recent study reported that MV of frontal and temporal cortex of subclinical AD and AD groups had significantly higher levels of col IV staining than a control group [18]. In contrast (and similar to our findings), Christov et al. reported that col I and III increased, but col IV decreased, in MV from AD subjects relative to MV from healthy controls [15]. Fibronectin is a large glycoprotein that exists in a soluble form in the plasma, and is a ubiquitous insoluble dimer in most tissues [56]. Recent IHC studies demonstrate that cellular fibronectin staining is highly variable, with an increase noted in frontal cortex, but not temporal cortex, in AD brains relative to age-matched controls [18]. These data are consistent with our observation, in western immunoblots, of no detectable differences in fibronectin content in MV isolated from high AD vs. not AD parietal cortex. We also did not find AD-related changes in the HSPG perlecan, which is present in low levels in MV [17]. Correspondingly, earlier investigations found no significant difference in perlecan mRNA levels in tissue of the hippocampus of AD patients when compared to age‐matched controls [57]. Recent IHC studies suggest perlecan and other HSPGs are increased in AD, which correlates with increased staining for Aβ [18, 58].

In contrast to the cases studied for ADNC, we found that laminin content was significantly higher in lysates of MV isolated from moderate-severe CAA vs. no CAA brains. We could not detect differences in col IV, perlecan, or fibronectin, which could reflect the limited number of CAA samples that were available. Of note, the higher level of MV-associated laminin in the presence of vascular Aβ deposition due to CAA is consistent with other processes that induce tension and BM thickening in vasculature [44, 59]. The presence of CAA may promote the deposition of ECM as a generalized response to stress on the BM. Conversely, we find microvasculature in the presence of high ADNC has a decrease in BM ECM content that could reflect a response to neuronal loss, pericyte drop-out and increased matrix metalloprotease activity [60, 61]. Conflicting results obtained in efforts to quantify changes in ECM associated with development of brain pathology likely reflect: (1) differences in ECM composition between the different brain regions that were sampled, (2) variations in the local microenvironment of plaques and tangles within a specific brain region, and (3) differences in the methods or techniques utilized to measure specific ECM components. We note our sample numbers were constrained by the requirement to have minimal other vascular or neurodegenerative processes, and our results are limited to analyses of portions of brain cortex that are representative of ADNC or CAA, respectively. Nonetheless, these results indicate that measures of BM content in MV will reflect the dominant pathology in any given section of brain tissue, and underscore the fact that brain MV will have unique ECM compositions that are determined by their microenvironment.

Unlike BM ECM components, both TSG-6 and HA content are greater in moderate-severe CAA relative to no CAA, similar to the higher levels we previously found in high ADNC relative to no ADNC. These results are not surprising—HA is a prevalent non-sulfated GAG that increases in content during CNS aging and injury, including neurodegenerative processes [13, 35]. Structural and functional effects of HA are largely mediated by the hyaladherins to which it is bound [21, 62]. In the brain, HA is most highly associated with chondroitin sulfate proteoglycans (CSPGs), but the inflammation-associated hyaladherin TSG-6 is also constitutively present, where it has been implicated in the organization of the HA matrix [27]. Complexes of HA and TSG-6 have enhanced binding to the HA receptor CD44 [29, 63]. This may explain how TSG-6 mediates some of its anti-inflammatory effects in a CD44 dependent manner [28, 64, 65]. Further studies are needed to determine whether the increase in HA and TSG-6 is a modulator in the microvascular response to AD and CAA, or represents a generalized response to injury.

## Conclusion

In conclusion, we find that in the absence of significant vascular disease, the brains of subjects with ADNC vs. not ADNC and CAA vs. no CAA did not differ with respect to the density and diameter of MV. However, MV-associated BM was altered with lower levels of both laminin and col IV in the BM of MV isolated from high ADNC vs. not ADNC tissues, but higher levels of laminin in the BM of MV isolated from moderate-severe CAA vs. no CAA brains. Similar to ADNC, CAA MV have a higher amount of TSG-6 and HA. These data demonstrate that the general morphology of brain MV is not appreciably altered following development of ADNC or CAA, but there are notable changes in BM ECM content and increases in prevalent ECM components like HA, which could have disease and regional specific effects on microvascular function.

## Data Availability

The datasets during and/or analysed during the current study available from the corresponding author on reasonable request.
